# Development of an Open Database of Genes Included in Hereditary Cancer Genetic Testing Panels Available From Major Sources in the US

**DOI:** 10.1001/jamaoncol.2021.7639

**Published:** 2022-02-03

**Authors:** Janis Geary, Mary Majumder, Christi Guerrini, Robert Cook-Deegan

**Affiliations:** 1 Barrett and O’Connor Washington Center, School for the Future of Innovation in Society, Consortium for Science, Policy, and Outcomes, Arizona State University, Washington, DC; 2 Center for Medical Ethics and Health Policy, Baylor College of Medicine, Houston, Texas

## Abstract

This quality improvement study describes the development of an open database of genes included in hereditary cancer genetic testing panels from major US providers and also discusses publicly available variant data for those genes.

Hereditary cancer syndromes occur when germline variants increase an individual’s risk of developing cancer. Genetic testing can identify these variants, enabling clinicians to intervene through increased screening or prophylactic surgery. Advances in DNA sequencing have driven a shift to sequence-based testing of multiple genes and to subsequent classification of the identified variants. This approach has generated copious data, but a serious problem remains: New variants in cancer genes are discovered daily that cannot be classified (scientifically or clinically). This problem of variants of unknown significance (VUSs) is exacerbated in groups with predominantly non-European ancestry who have not been included in data sets used to interpret variants because of underrepresentation in genetic studies and diminished access to clinical genetic testing.^1^ Fewer data lead to a higher proportion of VUSs.^2^ Policies for data sharing and the related infrastructure are emerging to support variant classifications.^3^ To inform these efforts, we have compiled a list of genes included in hereditary cancer genetic testing panels. We also describe publicly available variant data for those genes. Our analysis identifies substantial variability across panels and gaps in available variant data that should be a focus for those engaged in building a robust system for interpreting inherited cancer risk.

## Methods

This quality improvement study was reviewed and approved by the Arizona State University Institutional Review Board. Informed consent was not possible because this study uses publicly available gene variant data that are not attached to any participants.

We identified 17 major hereditary cancer testing companies and extracted data on their available hereditary cancer panels. We compiled a list of the genes included in at least 1 panel, and we extracted data on them from the ClinVar public database^4^ in October 2020. We also compiled information on genes included in clinical guidelines for hereditary cancer management and variants evaluated through the Clinical Genome Resource (ClinGen).^5^ We used Gephi software to display the overlap among companies and the genes offered in their hereditary cancer panels. In addition, we used Stata (StataCorp LLC) to perform descriptive statistical analyses. The complete data set is registered with the Open Science Framework.^6^

## Results

A total of 706 genes were included in at least 1 laboratory’s panel. Only 13 genes were included by all 17 companies. Only 110 genes appeared in at least 1 clinical guideline for hereditary cancer or had a ClinGen gene-disease relationship assessment. The Table provides summary statistics of variant-related characteristics available from ClinVar. The 699 genes with variants reported in ClinVar had a mean of 362 variants (median, 100 [range, 9-12 699]). The proportion of VUSs was a mean of 27.5% per gene, and 51.8% of variants per gene were provided by a single submitter. Genes included in clinical guidelines reported more variants, were included in more testing panels, had a higher proportion of variants identified during clinical testing, and had a higher proportion of VUSs. The Figure illustrates the considerable variability with which companies include genes in their panels, among a subset of laboratories submitting the most data to ClinVar.

**Table.** Available Variant Information for 699 Genes With Variants in ClinVar as of October 2020, With Genes Included or Not in Hereditary Cancer Clinical Guidelines^a^

	All genes in ClinVar (N = 699)	Genes included in guidelines (n = 110)^b^	Genes not included in guidelines (n = 589)
Per gene			
Mean No. of variants in ClinVar (range)	362 (9-12 699)	1352 (11-12 699)	177 (9-5580)
Mean No. of companies including it in a hereditary cancer panel (range)^c^	3.00 (1-15)	9.35 (1-17)	1.82 (1-17)
Variant-related information from ClinVar,^d^ %			
Method of variant data collection^e^			
Research	1.36	1.44	1.34
Literature only	5.49	4.92	5.59
Clinical testing	51.3	79.0	46.1
Clinical significance of variants			
Pathogenic or likely pathogenic	43.9	24.3	47.5
Benign or likely benign	26.3	26.2	26.3
Variant of unknown significance	27.5	46.8	23.9
Risk factor	0.24	0.08	0.27
Conflicting interpretations	2.01	3.74	2.06
Review status of variants^f^			
Practice guideline^g^	0.00	NA	0.00
Expert panel	0.48	1.35	0.31
Multiple submitters	6.53	16.4	4.68
Single submitter	51.84	62.9	49.8
At least 1 star	60.8	83.8	56.5

^a^ Excludes the 7 genes that had no variants in the ClinVar public database as of October 2020.

^b^ Guidelines refer to National Comprehensive Cancer Network (NCCN) guidelines (Genetic/Familial High-Risk Assessment of Breast, Ovarian, and Pancreatic Cancers; Genetic/Familial High-Risk Assessment of Colorectal Cancers; and other NCCN guidelines that include recommendations for assessing hereditary cancer risks), the International Cancer of the Pancreas Screening Consortium guidelines, the US Multi-Society Task Force on Colorectal Cancer guidelines, and the evaluations of gene-disease associations conducted by the Clinical Genome Resource (ClinGen). NA, not applicable.

^c^ We counted the number of companies that included each gene in at least 1 of their hereditary cancer test panels, and we report the mean number of companies. These results show that most genes are only tested for by few companies, with little overlap among companies. In addition, genes included in clinical guidelines are generally included by more companies.

^d^ Proportions are the number of variants for each classification divided by the total number of variants in ClinVar for each gene.

^e^ ClinVar uses the method of variant data collection as a variable that refers to the method used to collect data for a submission. The term *research* refers to variants interpreted as part of a research study, *literature only* refers to variants extracted from published literature, and *clinical testing* refers to variants that were interpreted as part of clinical genetic testing. Details of these definitions are available in the ClinVar Data Dictionary (https://www.ncbi.nlm.nih.gov/projects/clinvar/ClinVarDataDictionary.pdf). These results highlight that the majority of variants are derived from clinical testing, which skews the available data to those groups who can access clinical testing. Note that not all variants have data on the method of data collection, so the totals do not sum to 100.

^f^ This category refers to the review status of each variant, indicating whether it has been clinically classified as benign, pathogenic, unknown, and so forth, compared with clinical guidelines and ClinGen that evaluate the relationship between genes and cancer syndromes. A review status of at least 1 star indicates that at a minimum, a single submitter provided an interpretation with assertion criteria and evidence or multiple submitters did the same but had conflicting interpretations. Review status definitions are available in the ClinVar Data Dictionary.

^g^ The only gene in our data set with any variants with a review status of practice guideline was *CFTR*; the 23 variants with practice guidelines are risk factors for cystic fibrosis, not any hereditary cancer.

**Figure.**
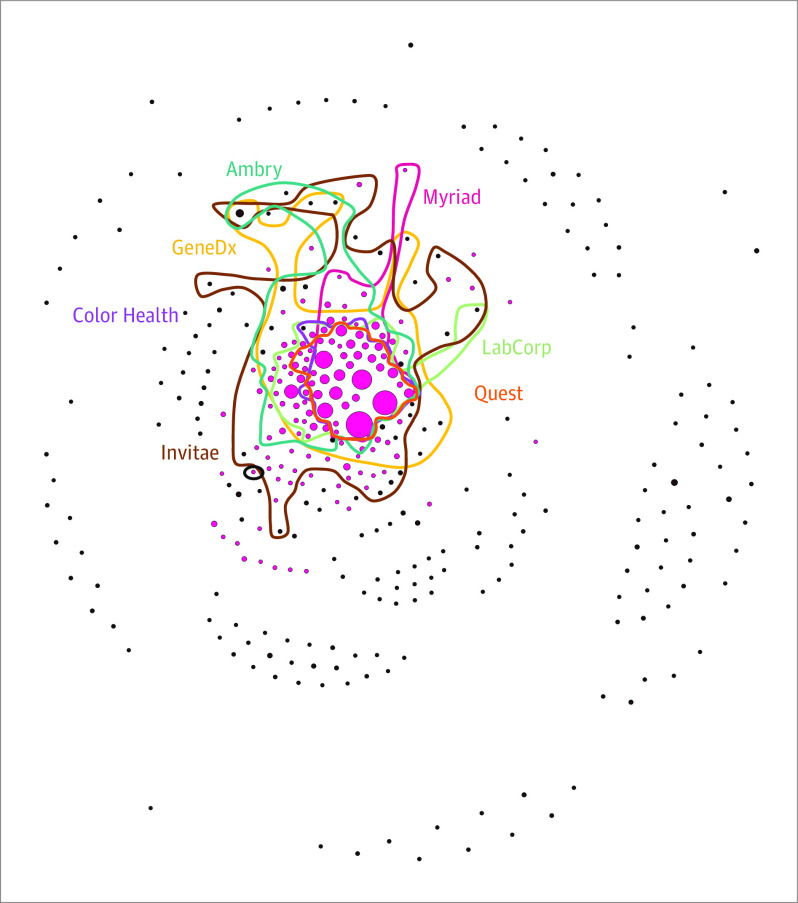
Network Depiction of Genes Appearing Together in a Test Panel, ClinGen Gene-Disease Evaluation, or Clinical Guideline The graph displays all 706 genes, with each gene appearing as a dot; the size of each dot corresponds to the number of variants for that gene in the ClinVar public database in October 2020 (the range of dot sizes is 5-40 and the range of number of variants is 9-12 699). Pink dots indicate that the gene has either had a Clinical Genome Resource (ClinGen) gene-disease evaluation or appears in clinical guidelines for hereditary cancer testing. The lines display the overlap among genes included in panels offered by 7 companies (Ambry, Color Health, Invitae, GeneDx, Labcorp, Myriad, and Quest). These companies were selected based on their submissions to ClinVar and their prominence in the genetic testing market. Genes in the interior of the graph are offered in the most testing panels, and those in the periphery are included in few panels. To note, even if the category name did not include the term *hereditary*, all panels were located on the section of the company’s website for hereditary cancer.

## Discussion

Multigene, sequence-based genetic testing for an inherited risk of cancer has increased dramatically since 2012. Our analysis suggests that (1) there is a lack of standards for which genes are offered in hereditary cancer panels, with companies offering tests for genes with very little publicly available variant data to support clinical interpretation, and (2) clinical guidelines are also lacking. We also describe the importance of clinical testing as a source of variant data and thus the intertwined nature of equitable access to clinical testing and the return of informative results. One limitation of this study is its cross-sectional design, which restricted our analysis of very dynamic data to what were publicly available at one time point.

We offer this description of the US hereditary cancer panel landscape as an empirical basis for ongoing policy discussions about how to improve data sharing to reduce the proportion of VUSs. We also demonstrate how analytical software and visualization tools can support future empirical work. The data are openly available and we welcome others to use these data and this landscape.^6^
